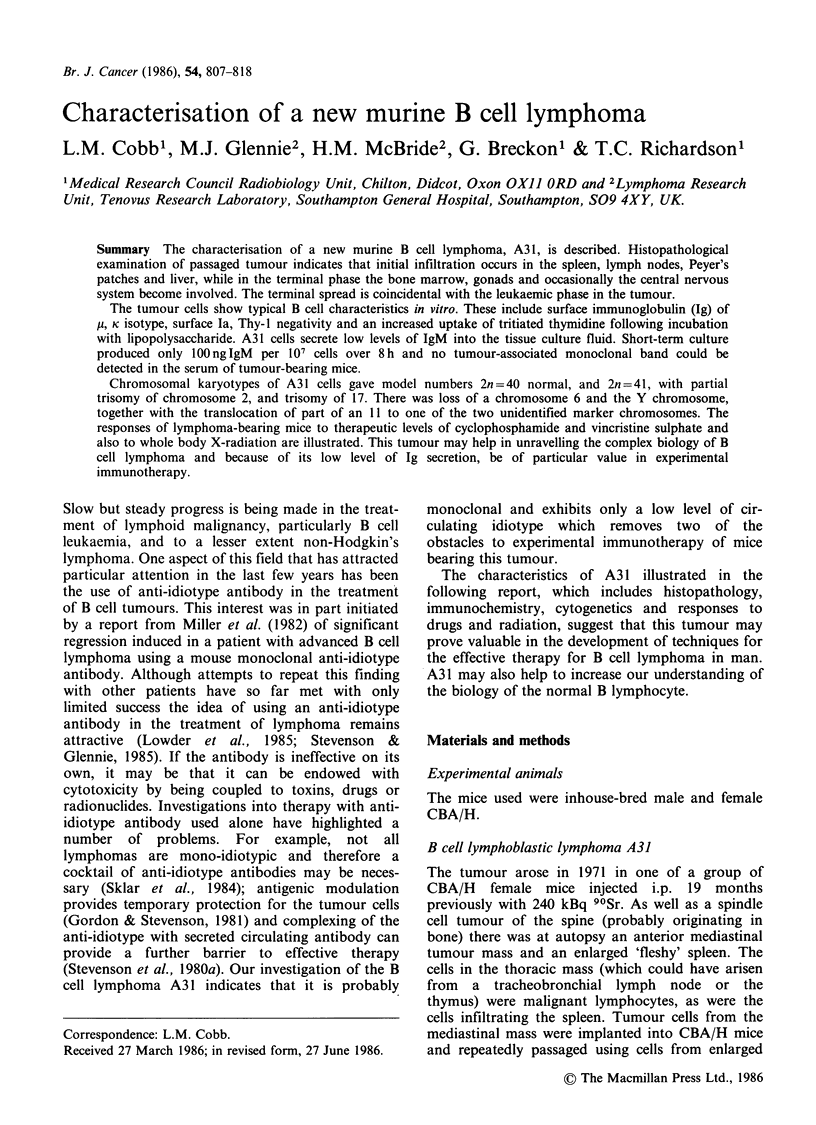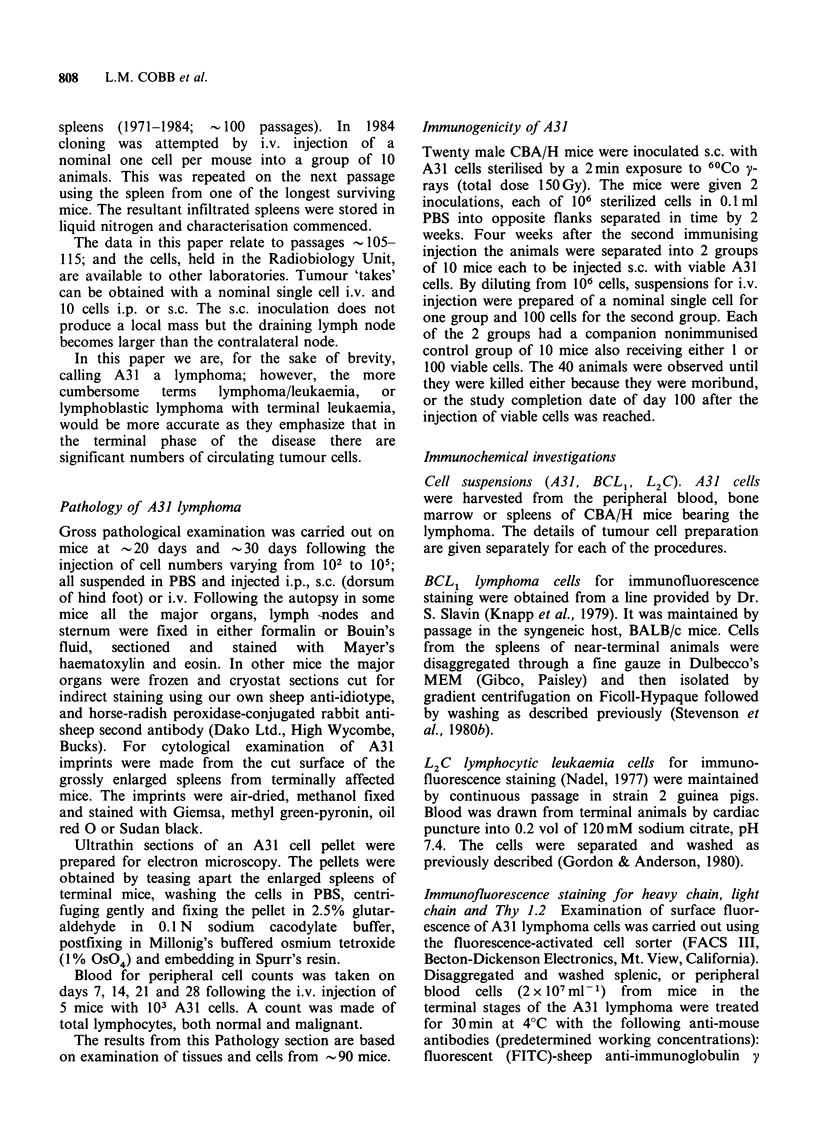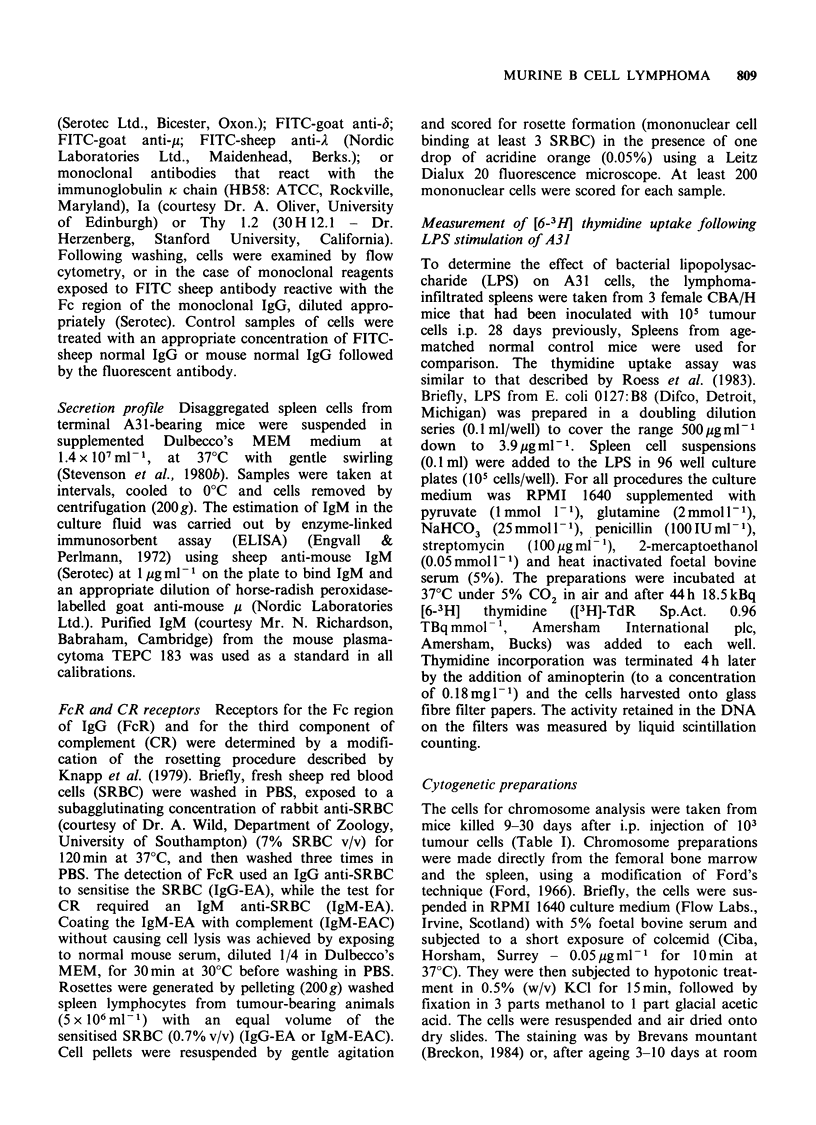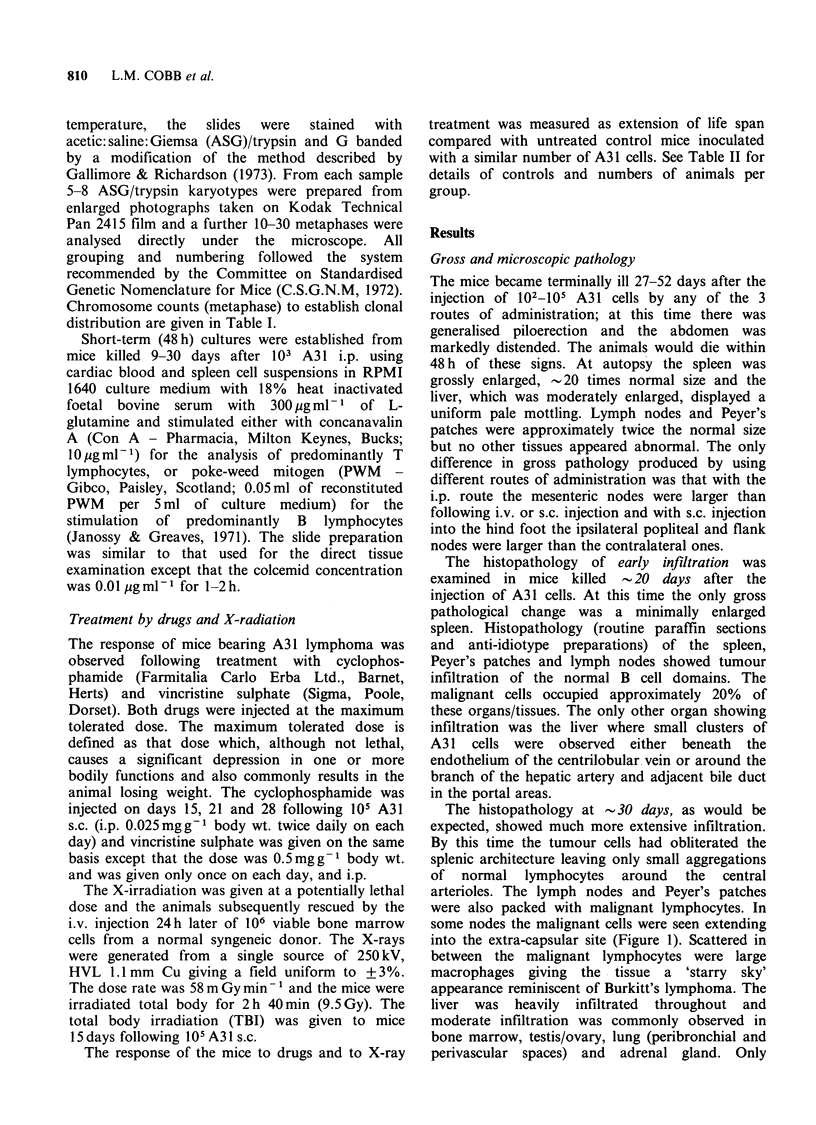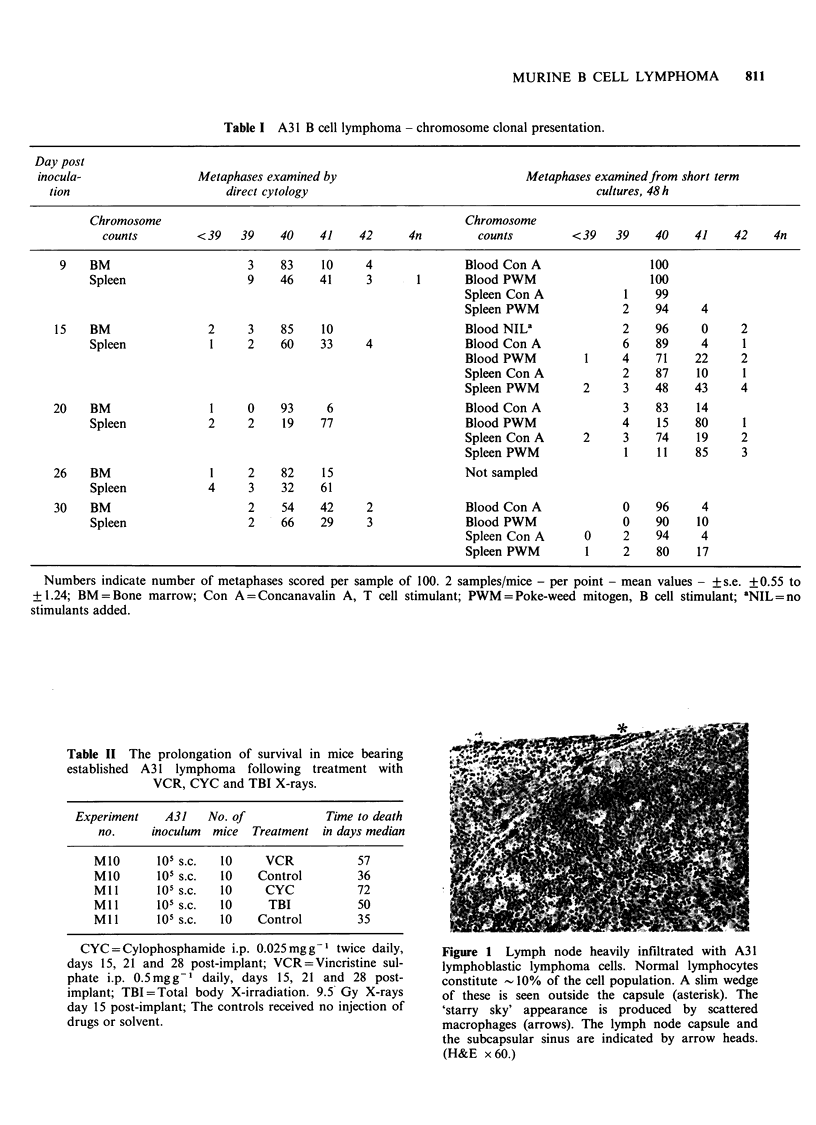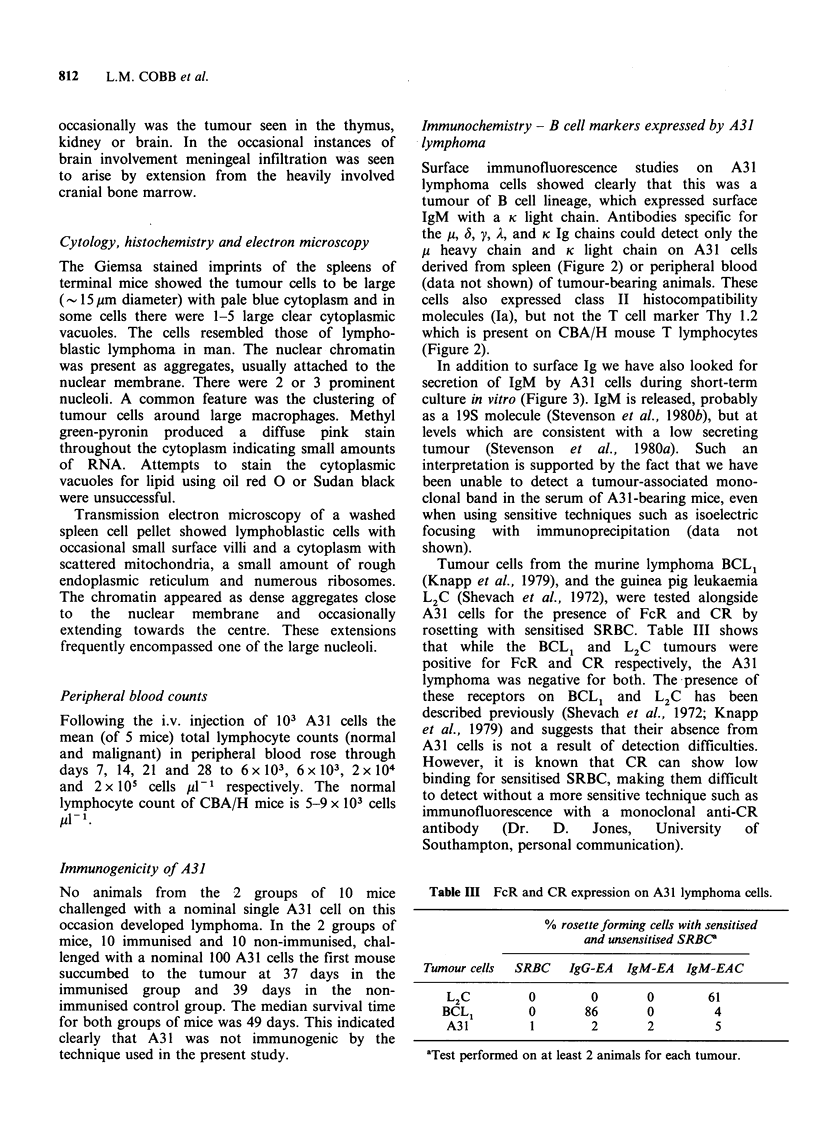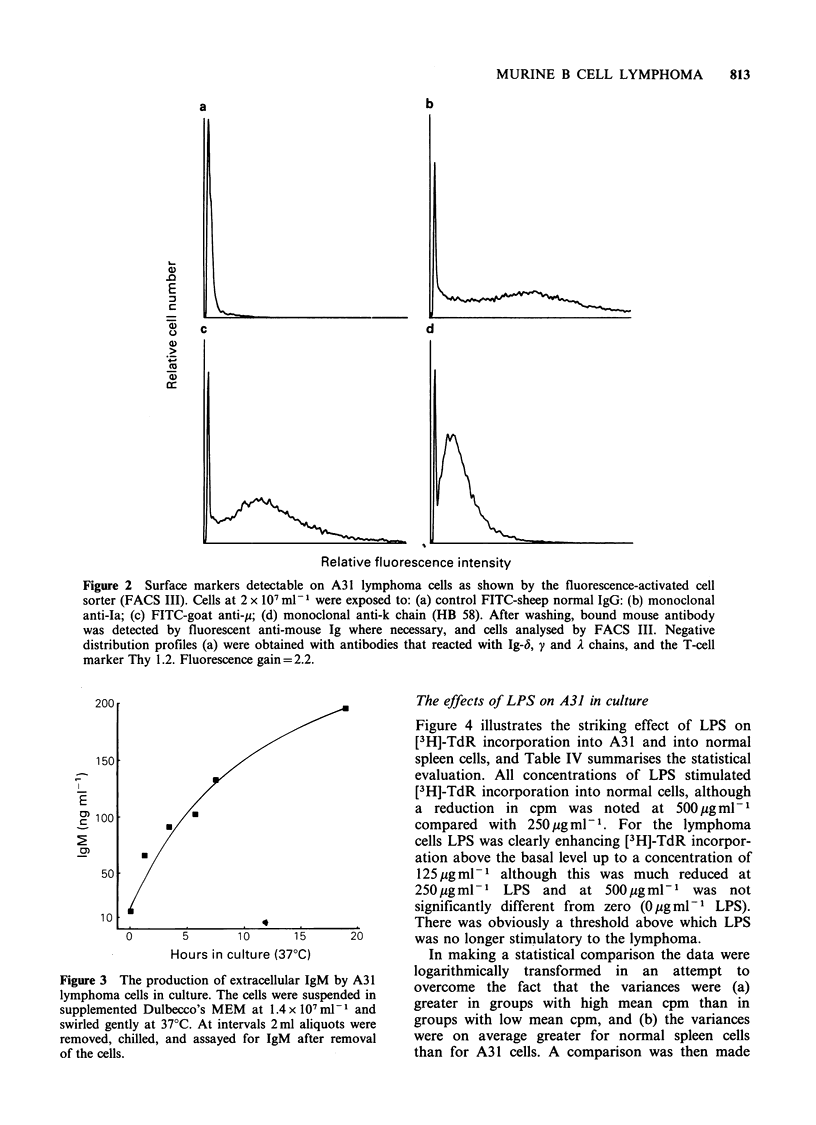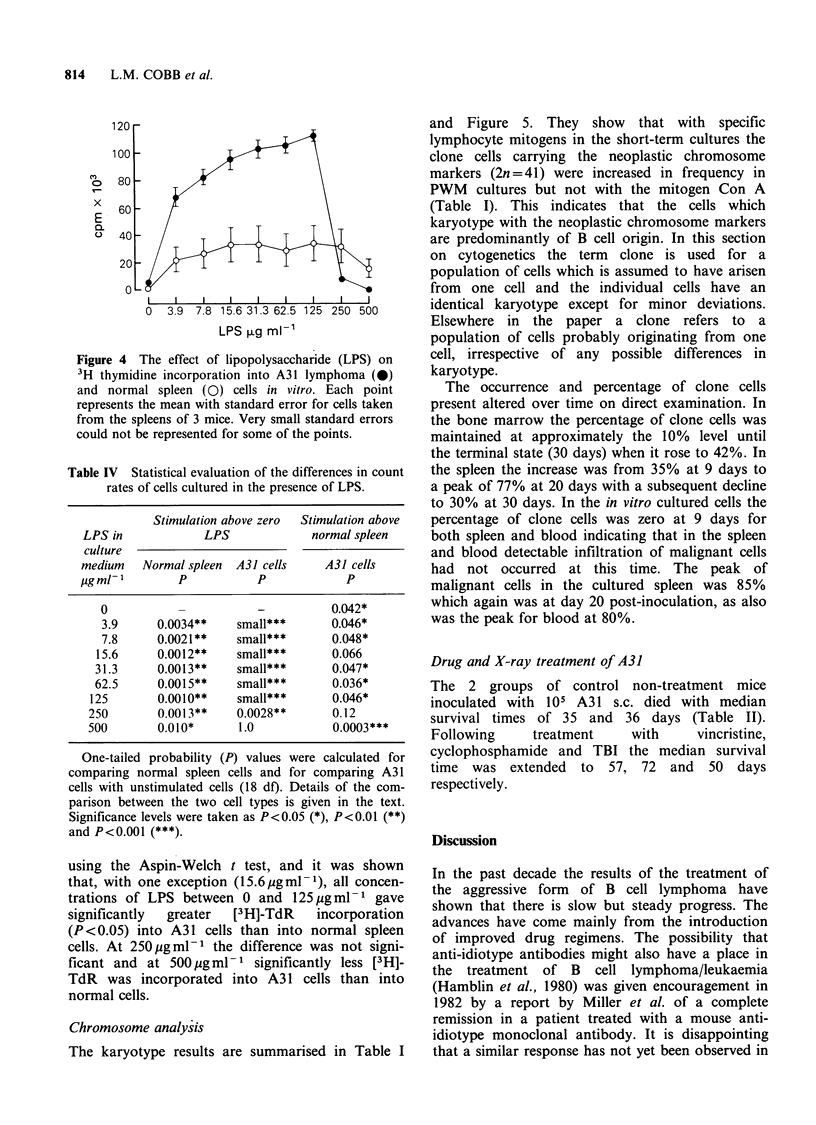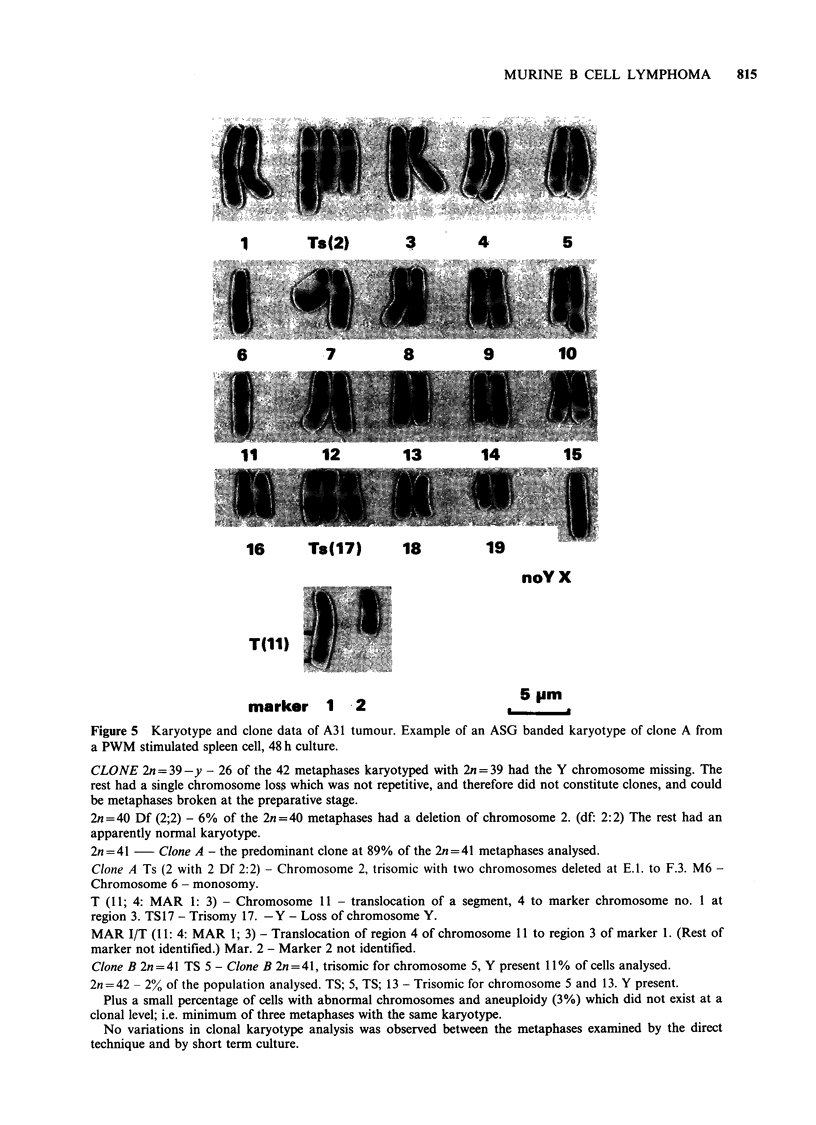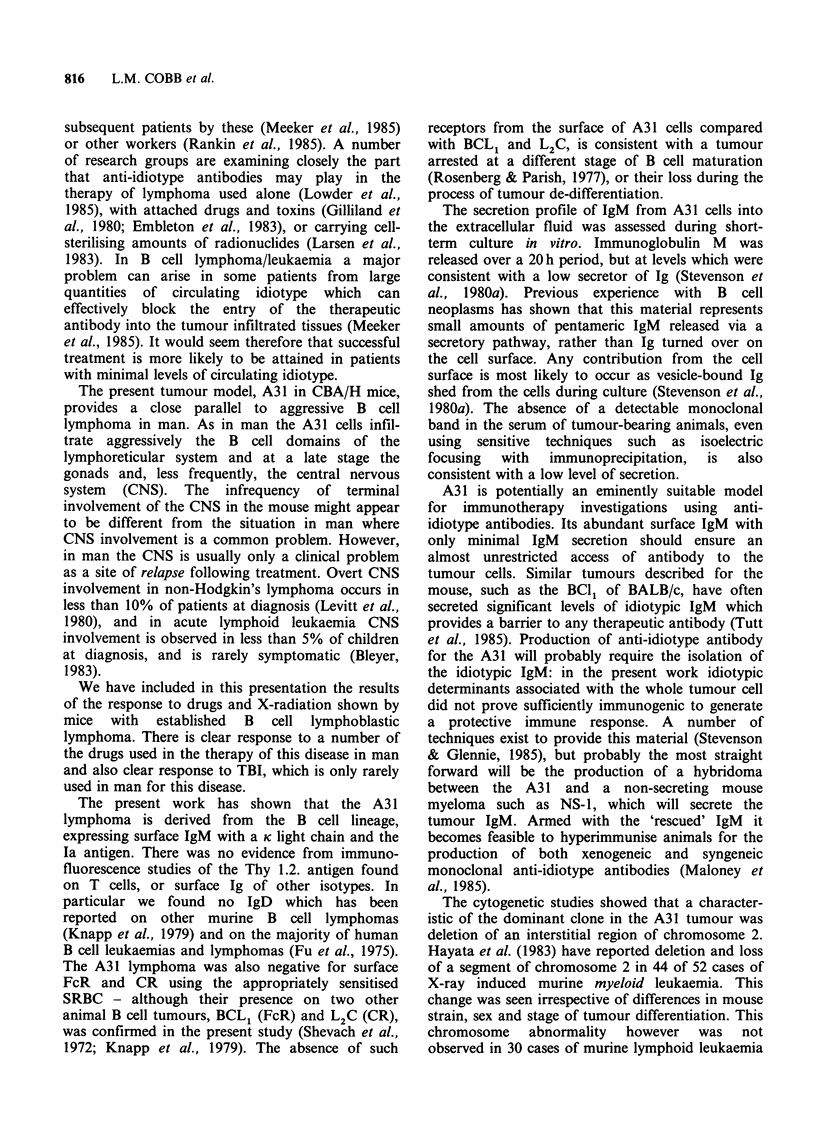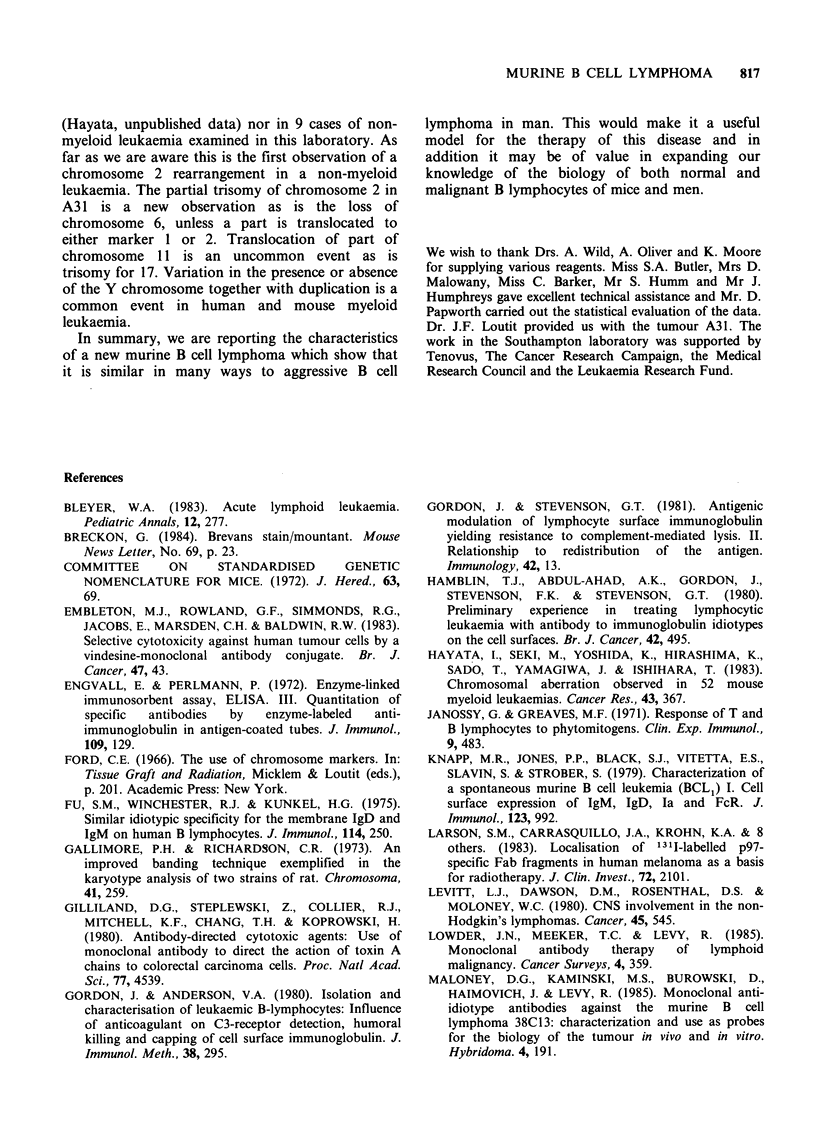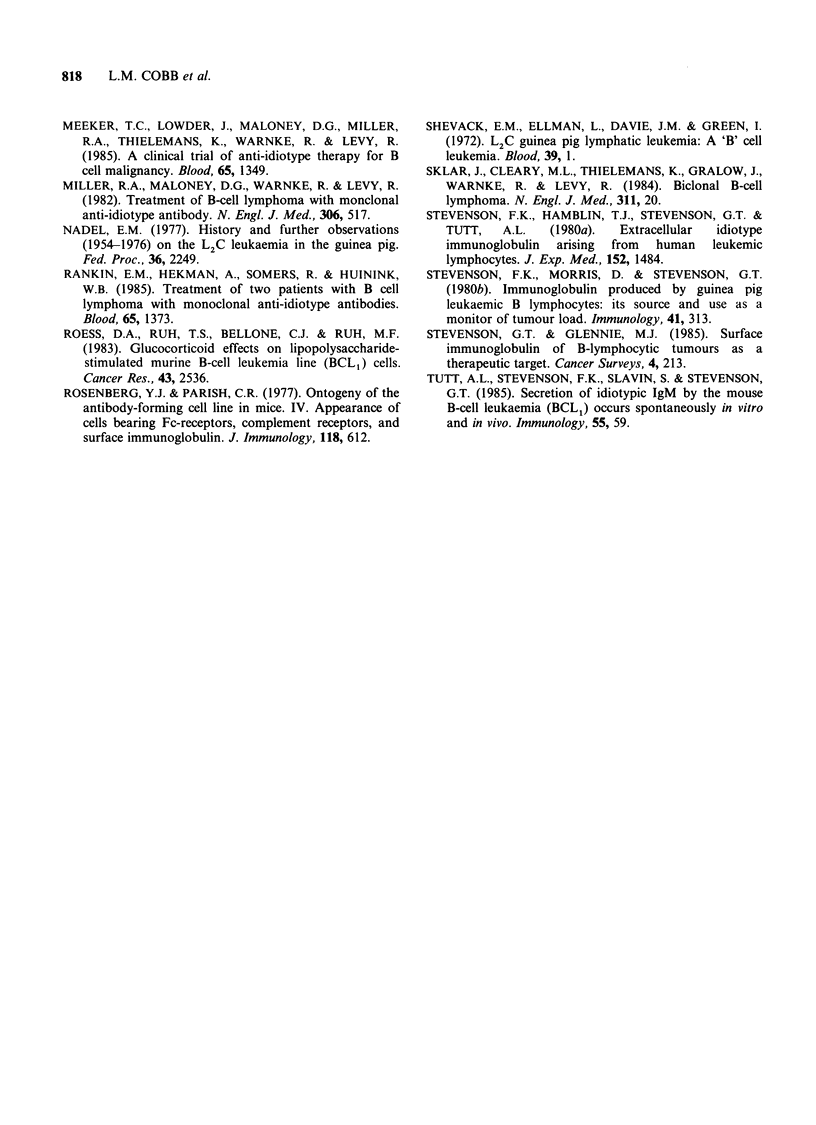# Characterisation of a new murine B cell lymphoma.

**DOI:** 10.1038/bjc.1986.244

**Published:** 1986-11

**Authors:** L. M. Cobb, M. J. Glennie, H. M. McBride, G. Breckon, T. C. Richardson

## Abstract

**Images:**


Br. J. Cancer (1986), 54, 807–818

### Characterisation of a new murine B cell lymphoma

L.M. Cobb[1], M.J. Glennie[2], H.M. McBride[2], G. Breckon[1] & T.C. Richardson[1]

[1]*Medical Research Council Radiobiology Unit, Chilton, Didcot, Oxon OX11 0RD and* [2]*Lymphoma Research Unit, Tenovus Research Laboratory, Southampton General Hospital, Southampton, SO9 4XY, UK.*

**Summary** The characterisation of a new murine B cell lymphoma, A31, is described. Histopathological examination of passaged tumour indicates that initial infiltration occurs in the spleen, lymph nodes, Peyer's patches and liver, while in the terminal phase the bone marrow, gonads and occasionally the central nervous system become involved. The terminal spread is coincidental with the leukaemic phase in the tumour.

The tumour cells show typical B cell characteristics *in vitro*. These include surface immunoglobulin (Ig) of $\mu$, $\kappa$ isotype, surface Ia, Thy-1 negativity and an increased uptake of tritiated thymidine following incubation with lipopolysaccharide. A31 cells secrete low levels of IgM into the tissue culture fluid. Short-term culture produced only 100 ng IgM per $10^7$ cells over 8 h and no tumour-associated monoclonal band could be detected in the serum of tumour-bearing mice.

Chromosomal karyotypes of A31 cells gave model numbers $2n=40$ normal, and $2n=41$, with partial trisomy of chromosome 2, and trisomy of 17. There was loss of a chromosome 6 and the Y chromosome, together with the translocation of part of an 11 to one of the two unidentified marker chromosomes. The responses of lymphoma-bearing mice to therapeutic levels of cyclophosphamide and vincristine sulphate and also to whole body X-radiation are illustrated. This tumour may help in unravelling the complex biology of B cell lymphoma and because of its low level of Ig secretion, be of particular value in experimental immunotherapy.

Slow but steady progress is being made in the treatment of lymphoid malignancy, particularly B cell leukaemia, and to a lesser extent non-Hodgkin's lymphoma. One aspect of this field that has attracted particular attention in the last few years has been the use of anti-idiotype antibody in the treatment of B cell tumours. This interest was in part initiated by a report from Miller *et al.* (1982) of significant regression induced in a patient with advanced B cell lymphoma using a mouse monoclonal anti-idiotype antibody. Although attempts to repeat this finding with other patients have so far met with only limited success the idea of using an anti-idiotype antibody in the treatment of lymphoma remains attractive (Lowder *et al.*, 1985; Stevenson & Glennie, 1985). If the antibody is ineffective on its own, it may be that it can be endowed with cytotoxicity by being coupled to toxins, drugs or radionuclides. Investigations into therapy with anti-idiotype antibody used alone have highlighted a number of problems. For example, not all lymphomas are mono-idiotypic and therefore a cocktail of anti-idiotype antibodies may be necessary (Sklar *et al.*, 1984); antigenic modulation provides temporary protection for the tumour cells (Gordon & Stevenson, 1981) and complexing of the anti-idiotype with secreted circulating antibody can provide a further barrier to effective therapy (Stevenson *et al.*, 1980*a*). Our investigation of the B cell lymphoma A31 indicates that it is probably monoclonal and exhibits only a low level of circulating idiotype which removes two of the obstacles to experimental immunotherapy of mice bearing this tumour.

The characteristics of A31 illustrated in the following report, which includes histopathology, immunochemistry, cytogenetics and responses to drugs and radiation, suggest that this tumour may prove valuable in the development of techniques for the effective therapy for B cell lymphoma in man. A31 may also help to increase our understanding of the biology of the normal B lymphocyte.

### Materials and methods

### Experimental animals

The mice used were inhouse-bred male and female CBA/H.

### B cell lymphoblastic lymphoma A31

The tumour arose in 1971 in one of a group of CBA/H female mice injected i.p. 19 months previously with 240 kBq $^{90}$Sr. As well as a spindle cell tumour of the spine (probably originating in bone) there was at autopsy an anterior mediastinal tumour mass and an enlarged 'fleshy' spleen. The cells in the thoracic mass (which could have arisen from a tracheobronchial lymph node or the thymus) were malignant lymphocytes, as were the cells infiltrating the spleen. Tumour cells from the mediastinal mass were implanted into CBA/H mice and repeatedly passaged using cells from enlarged

Correspondence: L.M. Cobb.
Received 27 March 1986; in revised form, 27 June 1986.

© The Macmillan Press Ltd., 1986

spleens (1971–1984; ~100 passages). In 1984 cloning was attempted by i.v. injection of a nominal one cell per mouse into a group of 10 animals. This was repeated on the next passage using the spleen from one of the longest surviving mice. The resultant infiltrated spleens were stored in liquid nitrogen and characterisation commenced.

The data in this paper relate to passages ~105–115; and the cells, held in the Radiobiology Unit, are available to other laboratories. Tumour 'takes' can be obtained with a nominal single cell i.v. and 10 cells i.p. or s.c. The s.c. inoculation does not produce a local mass but the draining lymph node becomes larger than the contralateral node.

In this paper we are, for the sake of brevity, calling A31 a lymphoma; however, the more cumbersome terms lymphoma/leukaemia, or lymphoblastic lymphoma with terminal leukaemia, would be more accurate as they emphasize that in the terminal phase of the disease there are significant numbers of circulating tumour cells.

*Pathology of A31 lymphoma*

Gross pathological examination was carried out on mice at ~20 days and ~30 days following the injection of cell numbers varying from $10^2$ to $10^5$; all suspended in PBS and injected i.p., s.c. (dorsum of hind foot) or i.v. Following the autopsy in some mice all the major organs, lymph nodes and sternum were fixed in either formalin or Bouin's fluid, sectioned and stained with Mayer's haematoxylin and eosin. In other mice the major organs were frozen and cryostat sections cut for indirect staining using our own sheep anti-idiotype, and horse-radish peroxidase-conjugated rabbit anti-sheep second antibody (Dako Ltd., High Wycombe, Bucks). For cytological examination of A31 imprints were made from the cut surface of the grossly enlarged spleens from terminally affected mice. The imprints were air-dried, methanol fixed and stained with Giemsa, methyl green-pyronin, oil red O or Sudan black.

Ultrathin sections of an A31 cell pellet were prepared for electron microscopy. The pellets were obtained by teasing apart the enlarged spleens of terminal mice, washing the cells in PBS, centrifuging gently and fixing the pellet in 2.5% glutaraldehyde in 0.1 N sodium cacodylate buffer, postfixing in Millonig's buffered osmium tetroxide (1% $OsO_4$) and embedding in Spurr's resin.

Blood for peripheral cell counts was taken on days 7, 14, 21 and 28 following the i.v. injection of 5 mice with $10^3$ A31 cells. A count was made of total lymphocytes, both normal and malignant.

The results from this Pathology section are based on examination of tissues and cells from ~90 mice.

*Immunogenicity of A31*

Twenty male CBA/H mice were inoculated s.c. with A31 cells sterilised by a 2 min exposure to $^{60}Co$ $\gamma$-rays (total dose 150 Gy). The mice were given 2 inoculations, each of $10^6$ sterilized cells in 0.1 ml PBS into opposite flanks separated in time by 2 weeks. Four weeks after the second immunising injection the animals were separated into 2 groups of 10 mice each to be injected s.c. with viable A31 cells. By diluting from $10^6$ cells, suspensions for i.v. injection were prepared of a nominal single cell for one group and 100 cells for the second group. Each of the 2 groups had a companion nonimmunised control group of 10 mice also receiving either 1 or 100 viable cells. The 40 animals were observed until they were killed either because they were moribund, or the study completion date of day 100 after the injection of viable cells was reached.

*Immunochemical investigations*

*Cell suspensions (A31, $BCL_1$, $L_2C$). A31 cells* were harvested from the peripheral blood, bone marrow or spleens of CBA/H mice bearing the lymphoma. The details of tumour cell preparation are given separately for each of the procedures.

*$BCL_1$ lymphoma cells* for immunofluorescence staining were obtained from a line provided by Dr. S. Slavin (Knapp *et al.*, 1979). It was maintained by passage in the syngeneic host, BALB/c mice. Cells from the spleens of near-terminal animals were disaggregated through a fine gauze in Dulbecco's MEM (Gibco, Paisley) and then isolated by gradient centrifugation on Ficoll-Hypaque followed by washing as described previously (Stevenson *et al.*, 1980*b*).

*$L_2C$ lymphocytic leukaemia cells* for immunofluorescence staining (Nadel, 1977) were maintained by continuous passage in strain 2 guinea pigs. Blood was drawn from terminal animals by cardiac puncture into 0.2 vol of 120 mM sodium citrate, pH 7.4. The cells were separated and washed as previously described (Gordon & Anderson, 1980).

*Immunofluorescence staining for heavy chain, light chain and Thy 1.2* Examination of surface fluorescence of A31 lymphoma cells was carried out using the fluorescence-activated cell sorter (FACS III, Becton-Dickenson Electronics, Mt. View, California). Disaggregated and washed splenic, or peripheral blood cells ($2 \times 10^7 ml^{-1}$) from mice in the terminal stages of the A31 lymphoma were treated for 30 min at 4°C with the following anti-mouse antibodies (predetermined working concentrations): fluorescent (FITC)-sheep anti-immunoglobulin $\gamma$

(Serotec Ltd., Bicester, Oxon.); FITC-goat anti-$\delta$; FITC-goat anti-$\mu$; FITC-sheep anti-$\lambda$ (Nordic Laboratories Ltd., Maidenhead, Berks.); or monoclonal antibodies that react with the immunoglobulin $\kappa$ chain (HB58: ATCC, Rockville, Maryland), Ia (courtesy Dr. A. Oliver, University of Edinburgh) or Thy 1.2 (30H12.1 – Dr. Herzenberg, Stanford University, California). Following washing, cells were examined by flow cytometry, or in the case of monoclonal reagents exposed to FITC sheep antibody reactive with the Fc region of the monoclonal IgG, diluted appropriately (Serotec). Control samples of cells were treated with an appropriate concentration of FITC-sheep normal IgG or mouse normal IgG followed by the fluorescent antibody.

*Secretion profile* Disaggregated spleen cells from terminal A31-bearing mice were suspended in supplemented Dulbecco's MEM medium at $1.4 \times 10^7 ml^{-1}$, at 37°C with gentle swirling (Stevenson *et al.*, 1980*b*). Samples were taken at intervals, cooled to 0°C and cells removed by centrifugation (200*g*). The estimation of IgM in the culture fluid was carried out by enzyme-linked immunosorbent assay (ELISA) (Engvall & Perlmann, 1972) using sheep anti-mouse IgM (Serotec) at $1 \mu g ml^{-1}$ on the plate to bind IgM and an appropriate dilution of horse-radish peroxidase-labelled goat anti-mouse $\mu$ (Nordic Laboratories Ltd.). Purified IgM (courtesy Mr. N. Richardson, Babraham, Cambridge) from the mouse plasmacytoma TEPC 183 was used as a standard in all calibrations.

*FcR and CR receptors* Receptors for the Fc region of IgG (FcR) and for the third component of complement (CR) were determined by a modification of the rosetting procedure described by Knapp *et al.* (1979). Briefly, fresh sheep red blood cells (SRBC) were washed in PBS, exposed to a subagglutinating concentration of rabbit anti-SRBC (courtesy of Dr. A. Wild, Department of Zoology, University of Southampton) (7% SRBC v/v) for 120 min at 37°C, and then washed three times in PBS. The detection of FcR used an IgG anti-SRBC to sensitise the SRBC (IgG-EA), while the test for CR required an IgM anti-SRBC (IgM-EA). Coating the IgM-EA with complement (IgM-EAC) without causing cell lysis was achieved by exposing to normal mouse serum, diluted 1/4 in Dulbecco's MEM, for 30 min at 30°C before washing in PBS. Rosettes were generated by pelleting (200*g*) washed spleen lymphocytes from tumour-bearing animals ($5 \times 10^6 ml^{-1}$) with an equal volume of the sensitised SRBC (0.7% v/v) (IgG-EA or IgM-EAC). Cell pellets were resuspended by gentle agitation and scored for rosette formation (mononuclear cell binding at least 3 SRBC) in the presence of one drop of acridine orange (0.05%) using a Leitz Dialux 20 fluorescence microscope. At least 200 mononuclear cells were scored for each sample.

*Measurement of [6-$^3$H] thymidine uptake following LPS stimulation of A31*

To determine the effect of bacterial lipopolysaccharide (LPS) on A31 cells, the lymphoma-infiltrated spleens were taken from 3 female CBA/H mice that had been inoculated with $10^5$ tumour cells i.p. 28 days previously, Spleens from age-matched normal control mice were used for comparison. The thymidine uptake assay was similar to that described by Roess *et al.* (1983). Briefly, LPS from E. coli 0127:B8 (Difco, Detroit, Michigan) was prepared in a doubling dilution series (0.1 ml/well) to cover the range $500 \mu g ml^{-1}$ down to $3.9 \mu g ml^{-1}$. Spleen cell suspensions (0.1 ml) were added to the LPS in 96 well culture plates ($10^5$ cells/well). For all procedures the culture medium was RPMI 1640 supplemented with pyruvate ($1 mmol\ l^{-1}$), glutamine ($2 mmol\ l^{-1}$), $NaHCO_3$ ($25 mmol\ l^{-1}$), penicillin ($100 IU ml^{-1}$), streptomycin ($100 \mu g ml^{-1}$), 2-mercaptoethanol ($0.05 mmol\ l^{-1}$) and heat inactivated foetal bovine serum (5%). The preparations were incubated at 37°C under 5% $CO_2$ in air and after 44 h 18.5 kBq [6-$^3$H] thymidine ([$^3$H]-TdR Sp.Act. 0.96 $TBq\ mmol^{-1}$, Amersham International plc, Amersham, Bucks) was added to each well. Thymidine incorporation was terminated 4 h later by the addition of aminopterin (to a concentration of $0.18 mg\ l^{-1}$) and the cells harvested onto glass fibre filter papers. The activity retained in the DNA on the filters was measured by liquid scintillation counting.

*Cytogenetic preparations*

The cells for chromosome analysis were taken from mice killed 9–30 days after i.p. injection of $10^3$ tumour cells (Table I). Chromosome preparations were made directly from the femoral bone marrow and the spleen, using a modification of Ford's technique (Ford, 1966). Briefly, the cells were suspended in RPMI 1640 culture medium (Flow Labs., Irvine, Scotland) with 5% foetal bovine serum and subjected to a short exposure of colcemid (Ciba, Horsham, Surrey – $0.05 \mu g ml^{-1}$ for 10 min at 37°C). They were then subjected to hypotonic treatment in 0.5% (w/v) KCl for 15 min, followed by fixation in 3 parts methanol to 1 part glacial acetic acid. The cells were resuspended and air dried onto dry slides. The staining was by Brevans mountant (Breckon, 1984) or, after ageing 3–10 days at room temperature, the slides were stained with acetic:saline:Giemsa (ASG)/trypsin and G banded by a modification of the method described by Gallimore & Richardson (1973). From each sample 5–8 ASG/trypsin karyotypes were prepared from enlarged photographs taken on Kodak Technical Pan 2415 film and a further 10–30 metaphases were analysed directly under the microscope. All grouping and numbering followed the system recommended by the Committee on Standardised Genetic Nomenclature for Mice (C.S.G.N.M, 1972). Chromosome counts (metaphase) to establish clonal distribution are given in Table I.

Short-term (48 h) cultures were established from mice killed 9–30 days after $10^3$ A31 i.p. using cardiac blood and spleen cell suspensions in RPMI 1640 culture medium with 18% heat inactivated foetal bovine serum with $300\,\mu g\,ml^{-1}$ of L-glutamine and stimulated either with concanavalin A (Con A – Pharmacia, Milton Keynes, Bucks; $10\,\mu g\,ml^{-1}$) for the analysis of predominantly T lymphocytes, or poke-weed mitogen (PWM – Gibco, Paisley, Scotland; 0.05 ml of reconstituted PWM per 5 ml of culture medium) for the stimulation of predominantly B lymphocytes (Janossy & Greaves, 1971). The slide preparation was similar to that used for the direct tissue examination except that the colcemid concentration was $0.01\,\mu g\,ml^{-1}$ for 1–2 h.

*Treatment by drugs and X-radiation*

The response of mice bearing A31 lymphoma was observed following treatment with cyclophosphamide (Farmitalia Carlo Erba Ltd., Barnet, Herts) and vincristine sulphate (Sigma, Poole, Dorset). Both drugs were injected at the maximum tolerated dose. The maximum tolerated dose is defined as that dose which, although not lethal, causes a significant depression in one or more bodily functions and also commonly results in the animal losing weight. The cyclophosphamide was injected on days 15, 21 and 28 following $10^5$ A31 s.c. (i.p. $0.025\,mg\,g^{-1}$ body wt. twice daily on each day) and vincristine sulphate was given on the same basis except that the dose was $0.5\,mg\,g^{-1}$ body wt. and was given only once on each day, and i.p.

The X-irradiation was given at a potentially lethal dose and the animals subsequently rescued by the i.v. injection 24 h later of $10^6$ viable bone marrow cells from a normal syngeneic donor. The X-rays were generated from a single source of 250 kV, HVL 1.1 mm Cu giving a field uniform to $\pm 3\%$. The dose rate was $58\,mGy\,min^{-1}$ and the mice were irradiated total body for 2 h 40 min (9.5 Gy). The total body irradiation (TBI) was given to mice 15 days following $10^5$ A31 s.c.

The response of the mice to drugs and to X-ray treatment was measured as extension of life span compared with untreated control mice inoculated with a similar number of A31 cells. See Table II for details of controls and numbers of animals per group.

### Results

*Gross and microscopic pathology*

The mice became terminally ill 27–52 days after the injection of $10^2$–$10^5$ A31 cells by any of the 3 routes of administration; at this time there was generalised piloerection and the abdomen was markedly distended. The animals would die within 48 h of these signs. At autopsy the spleen was grossly enlarged, ~20 times normal size and the liver, which was moderately enlarged, displayed a uniform pale mottling. Lymph nodes and Peyer's patches were approximately twice the normal size but no other tissues appeared abnormal. The only difference in gross pathology produced by using different routes of administration was that with the i.p. route the mesenteric nodes were larger than following i.v. or s.c. injection and with s.c. injection into the hind foot the ipsilateral popliteal and flank nodes were larger than the contralateral ones.

The histopathology of *early infiltration* was examined in mice killed *~20 days* after the injection of A31 cells. At this time the only gross pathological change was a minimally enlarged spleen. Histopathology (routine paraffin sections and anti-idiotype preparations) of the spleen, Peyer's patches and lymph nodes showed tumour infiltration of the normal B cell domains. The malignant cells occupied approximately 20% of these organs/tissues. The only other organ showing infiltration was the liver where small clusters of A31 cells were observed either beneath the endothelium of the centrilobular vein or around the branch of the hepatic artery and adjacent bile duct in the portal areas.

The histopathology at *~30 days,* as would be expected, showed much more extensive infiltration. By this time the tumour cells had obliterated the splenic architecture leaving only small aggregations of normal lymphocytes around the central arterioles. The lymph nodes and Peyer's patches were also packed with malignant lymphocytes. In some nodes the malignant cells were seen extending into the extra-capsular site (Figure 1). Scattered in between the malignant lymphocytes were large macrophages giving the tissue a 'starry sky' appearance reminiscent of Burkitt's lymphoma. The liver was heavily infiltrated throughout and moderate infiltration was commonly observed in bone marrow, testis/ovary, lung (peribronchial and perivascular spaces) and adrenal gland. Only

**Table I** A31 B cell lymphoma – chromosome clonal presentation.

| *Day post inoculation* | *Metaphases examined by direct cytology* | | | | | | | *Metaphases examined from short term cultures, 48 h* | | | | | | |
|---|---|---|---|---|---|---|---|---|---|---|---|---|---|---|
| | *Chromosome counts* | *<39* | *39* | *40* | *41* | *42* | *4n* | *Chromosome counts* | *<39* | *39* | *40* | *41* | *42* | *4n* |
| 9 | BM | | 3 | 83 | 10 | 4 | | Blood Con A | | | 100 | | | |
| | Spleen | | 9 | 46 | 41 | 3 | 1 | Blood PWM | | | 100 | | | |
| | | | | | | | | Spleen Con A | | 1 | 99 | | | |
| | | | | | | | | Spleen PWM | | 2 | 94 | 4 | | |
| 15 | BM | 2 | 3 | 85 | 10 | | | Blood NIL[a] | | 2 | 96 | 0 | 2 | |
| | Spleen | 1 | 2 | 60 | 33 | 4 | | Blood Con A | | 6 | 89 | 4 | 1 | |
| | | | | | | | | Blood PWM | 1 | 4 | 71 | 22 | 2 | |
| | | | | | | | | Spleen Con A | | 2 | 87 | 10 | 1 | |
| | | | | | | | | Spleen PWM | 2 | 3 | 48 | 43 | 4 | |
| 20 | BM | 1 | 0 | 93 | 6 | | | Blood Con A | | 3 | 83 | 14 | | |
| | Spleen | 2 | 2 | 19 | 77 | | | Blood PWM | | 4 | 15 | 80 | 1 | |
| | | | | | | | | Spleen Con A | 2 | 3 | 74 | 19 | 2 | |
| | | | | | | | | Spleen PWM | | 1 | 11 | 85 | 3 | |
| 26 | BM | 1 | 2 | 82 | 15 | | | Not sampled | | | | | | |
| | Spleen | 4 | 3 | 32 | 61 | | | | | | | | | |
| 30 | BM | | 2 | 54 | 42 | 2 | | Blood Con A | | 0 | 96 | 4 | | |
| | Spleen | | 2 | 66 | 29 | 3 | | Blood PWM | | 0 | 90 | 10 | | |
| | | | | | | | | Spleen Con A | 0 | 2 | 94 | 4 | | |
| | | | | | | | | Spleen PWM | 1 | 2 | 80 | 17 | | |

Numbers indicate number of metaphases scored per sample of 100. 2 samples/mice – per point – mean values – ±s.e. ±0.55 to ±1.24; BM = Bone marrow; Con A = Concanavalin A, T cell stimulant; PWM = Poke-weed mitogen, B cell stimulant; [a]NIL = no stimulants added.

**Table II** The prolongation of survival in mice bearing established A31 lymphoma following treatment with VCR, CYC and TBI X-rays.

| *Experiment no.* | *A31 inoculum* | *No. of mice* | *Treatment* | *Time to death in days median* |
|---|---|---|---|---|
| M10 | $10^5$ s.c. | 10 | VCR | 57 |
| M10 | $10^5$ s.c. | 10 | Control | 36 |
| M11 | $10^5$ s.c. | 10 | CYC | 72 |
| M11 | $10^5$ s.c. | 10 | TBI | 50 |
| M11 | $10^5$ s.c. | 10 | Control | 35 |

CYC = Cylophosphamide i.p. $0.025 \mathrm{mg\,g}^{-1}$ twice daily, days 15, 21 and 28 post-implant; VCR = Vincristine sulphate i.p. $0.5 \mathrm{mg\,g}^{-1}$ daily, days 15, 21 and 28 post-implant; TBI = Total body X-irradiation. 9.5 Gy X-rays day 15 post-implant; The controls received no injection of drugs or solvent.

**Figure 1** Lymph node heavily infiltrated with A31 lymphoblastic lymphoma cells. Normal lymphocytes constitute ~10% of the cell population. A slim wedge of these is seen outside the capsule (asterisk). The 'starry sky' appearance is produced by scattered macrophages (arrows). The lymph node capsule and the subcapsular sinus are indicated by arrow heads. (H&E ×60.)

occasionally was the tumour seen in the thymus, kidney or brain. In the occasional instances of brain involvement meningeal infiltration was seen to arise by extension from the heavily involved cranial bone marrow.

*Cytology, histochemistry and electron microscopy*

The Giemsa stained imprints of the spleens of terminal mice showed the tumour cells to be large ($\sim 15\,\mu m$ diameter) with pale blue cytoplasm and in some cells there were 1–5 large clear cytoplasmic vacuoles. The cells resembled those of lymphoblastic lymphoma in man. The nuclear chromatin was present as aggregates, usually attached to the nuclear membrane. There were 2 or 3 prominent nucleoli. A common feature was the clustering of tumour cells around large macrophages. Methyl green-pyronin produced a diffuse pink stain throughout the cytoplasm indicating small amounts of RNA. Attempts to stain the cytoplasmic vacuoles for lipid using oil red O or Sudan black were unsuccessful.

Transmission electron microscopy of a washed spleen cell pellet showed lymphoblastic cells with occasional small surface villi and a cytoplasm with scattered mitochondria, a small amount of rough endoplasmic reticulum and numerous ribosomes. The chromatin appeared as dense aggregates close to the nuclear membrane and occasionally extending towards the centre. These extensions frequently encompassed one of the large nucleoli.

*Peripheral blood counts*

Following the i.v. injection of $10^3$ A31 cells the mean (of 5 mice) total lymphocyte counts (normal and malignant) in peripheral blood rose through days 7, 14, 21 and 28 to $6 \times 10^3$, $6 \times 10^3$, $2 \times 10^4$ and $2 \times 10^5$ cells $\mu l^{-1}$ respectively. The normal lymphocyte count of CBA/H mice is $5\text{–}9 \times 10^3$ cells $\mu l^{-1}$.

*Immunogenicity of A31*

No animals from the 2 groups of 10 mice challenged with a nominal single A31 cell on this occasion developed lymphoma. In the 2 groups of mice, 10 immunised and 10 non-immunised, challenged with a nominal 100 A31 cells the first mouse succumbed to the tumour at 37 days in the immunised group and 39 days in the non-immunised control group. The median survival time for both groups of mice was 49 days. This indicated clearly that A31 was not immunogenic by the technique used in the present study.

*Immunochemistry – B cell markers expressed by A31 lymphoma*

Surface immunofluorescence studies on A31 lymphoma cells showed clearly that this was a tumour of B cell lineage, which expressed surface IgM with a $\kappa$ light chain. Antibodies specific for the $\mu$, $\delta$, $\gamma$, $\lambda$, and $\kappa$ Ig chains could detect only the $\mu$ heavy chain and $\kappa$ light chain on A31 cells derived from spleen (Figure 2) or peripheral blood (data not shown) of tumour-bearing animals. These cells also expressed class II histocompatibility molecules (Ia), but not the T cell marker Thy 1.2 which is present on CBA/H mouse T lymphocytes (Figure 2).

In addition to surface Ig we have also looked for secretion of IgM by A31 cells during short-term culture *in vitro* (Figure 3). IgM is released, probably as a 19S molecule (Stevenson *et al.*, 1980*b*), but at levels which are consistent with a low secreting tumour (Stevenson *et al.*, 1980*a*). Such an interpretation is supported by the fact that we have been unable to detect a tumour-associated monoclonal band in the serum of A31-bearing mice, even when using sensitive techniques such as isoelectric focusing with immunoprecipitation (data not shown).

Tumour cells from the murine lymphoma $BCL_1$ (Knapp *et al.*, 1979), and the guinea pig leukaemia $L_2C$ (Shevach *et al.*, 1972), were tested alongside A31 cells for the presence of FcR and CR by rosetting with sensitised SRBC. Table III shows that while the $BCL_1$ and $L_2C$ tumours were positive for FcR and CR respectively, the A31 lymphoma was negative for both. The presence of these receptors on $BCL_1$ and $L_2C$ has been described previously (Shevach *et al.*, 1972; Knapp *et al.*, 1979) and suggests that their absence from A31 cells is not a result of detection difficulties. However, it is known that CR can show low binding for sensitised SRBC, making them difficult to detect without a more sensitive technique such as immunofluorescence with a monoclonal anti-CR antibody (Dr. D. Jones, University of Southampton, personal communication).

**Table III** FcR and CR expression on A31 lymphoma cells.

| | % rosette forming cells with sensitised and unsensitised SRBC[a] | | | |
|---|---|---|---|---|
| *Tumour cells* | *SRBC* | *IgG-EA* | *IgM-EA* | *IgM-EAC* |
| $L_2C$ | 0 | 0 | 0 | 61 |
| $BCL_1$ | 0 | 86 | 0 | 4 |
| A31 | 1 | 2 | 2 | 5 |

[a]Test performed on at least 2 animals for each tumour.

**Figure 2** Surface markers detectable on A31 lymphoma cells as shown by the fluorescence-activated cell sorter (FACS III). Cells at $2 \times 10^7 ml^{-1}$ were exposed to: (a) control FITC-sheep normal IgG: (b) monoclonal anti-Ia; (c) FITC-goat anti-$\mu$; (d) monoclonal anti-k chain (HB 58). After washing, bound mouse antibody was detected by fluorescent anti-mouse Ig where necessary, and cells analysed by FACS III. Negative distribution profiles (a) were obtained with antibodies that reacted with Ig-$\delta$, $\gamma$ and $\lambda$ chains, and the T-cell marker Thy 1.2. Fluorescence gain = 2.2.

**Figure 3** The production of extracellular IgM by A31 lymphoma cells in culture. The cells were suspended in supplemented Dulbecco's MEM at $1.4 \times 10^7 ml^{-1}$ and swirled gently at 37°C. At intervals 2 ml aliquots were removed, chilled, and assayed for IgM after removal of the cells.

*The effects of LPS on A31 in culture*

Figure 4 illustrates the striking effect of LPS on [$^3$H]-TdR incorporation into A31 and into normal spleen cells, and Table IV summarises the statistical evaluation. All concentrations of LPS stimulated [$^3$H]-TdR incorporation into normal cells, although a reduction in cpm was noted at $500 \mu g ml^{-1}$ compared with $250 \mu g ml^{-1}$. For the lymphoma cells LPS was clearly enhancing [$^3$H]-TdR incorporation above the basal level up to a concentration of $125 \mu g ml^{-1}$ although this was much reduced at $250 \mu g ml^{-1}$ LPS and at $500 \mu g ml^{-1}$ was not significantly different from zero ($0 \mu g ml^{-1}$ LPS). There was obviously a threshold above which LPS was no longer stimulatory to the lymphoma.

In making a statistical comparison the data were logarithmically transformed in an attempt to overcome the fact that the variances were (a) greater in groups with high mean cpm than in groups with low mean cpm, and (b) the variances were on average greater for normal spleen cells than for A31 cells. A comparison was then made

**Figure 4** The effect of lipopolysaccharide (LPS) on $^3H$ thymidine incorporation into A31 lymphoma (●) and normal spleen (○) cells *in vitro*. Each point represents the mean with standard error for cells taken from the spleens of 3 mice. Very small standard errors could not be represented for some of the points.

**Table IV** Statistical evaluation of the differences in count rates of cells cultured in the presence of LPS.

| *LPS in culture medium* $\mu g\,ml^{-1}$ | *Stimulation above zero LPS* *Normal spleen P* | *A31 cells P* | *Stimulation above normal spleen* *A31 cells P* |
|---|---|---|---|
| 0 | – | – | 0.042* |
| 3.9 | 0.0034** | small*** | 0.046* |
| 7.8 | 0.0021** | small*** | 0.048* |
| 15.6 | 0.0012** | small*** | 0.066 |
| 31.3 | 0.0013** | small*** | 0.047* |
| 62.5 | 0.0015** | small*** | 0.036* |
| 125 | 0.0010** | small*** | 0.046* |
| 250 | 0.0013** | 0.0028** | 0.12 |
| 500 | 0.010* | 1.0 | 0.0003*** |

One-tailed probability (*P*) values were calculated for comparing normal spleen cells and for comparing A31 cells with unstimulated cells (18 df). Details of the comparison between the two cell types is given in the text. Significance levels were taken as $P<0.05$ (*), $P<0.01$ (**) and $P<0.001$ (***).

using the Aspin-Welch *t* test, and it was shown that, with one exception ($15.6\,\mu g\,ml^{-1}$), all concentrations of LPS between 0 and $125\,\mu g\,ml^{-1}$ gave significantly greater [$^3H$]-TdR incorporation ($P<0.05$) into A31 cells than into normal spleen cells. At $250\,\mu g\,ml^{-1}$ the difference was not significant and at $500\,\mu g\,ml^{-1}$ significantly less [$^3H$]-TdR was incorporated into A31 cells than into normal cells.

*Chromosome analysis*

The karyotype results are summarised in Table I and Figure 5. They show that with specific lymphocyte mitogens in the short-term cultures the clone cells carrying the neoplastic chromosome markers ($2n=41$) were increased in frequency in PWM cultures but not with the mitogen Con A (Table I). This indicates that the cells which karyotype with the neoplastic chromosome markers are predominantly of B cell origin. In this section on cytogenetics the term clone is used for a population of cells which is assumed to have arisen from one cell and the individual cells have an identical karyotype except for minor deviations. Elsewhere in the paper a clone refers to a population of cells probably originating from one cell, irrespective of any possible differences in karyotype.

The occurrence and percentage of clone cells present altered over time on direct examination. In the bone marrow the percentage of clone cells was maintained at approximately the 10% level until the terminal state (30 days) when it rose to 42%. In the spleen the increase was from 35% at 9 days to a peak of 77% at 20 days with a subsequent decline to 30% at 30 days. In the *in vitro* cultured cells the percentage of clone cells was zero at 9 days for both spleen and blood indicating that in the spleen and blood detectable infiltration of malignant cells had not occurred at this time. The peak of malignant cells in the cultured spleen was 85% which again was at day 20 post-inoculation, as also was the peak for blood at 80%.

*Drug and X-ray treatment of A31*

The 2 groups of control non-treatment mice inoculated with $10^5$ A31 s.c. died with median survival times of 35 and 36 days (Table II). Following treatment with vincristine, cyclophosphamide and TBI the median survival time was extended to 57, 72 and 50 days respectively.

### Discussion

In the past decade the results of the treatment of the aggressive form of B cell lymphoma have shown that there is slow but steady progress. The advances have come mainly from the introduction of improved drug regimens. The possibility that anti-idiotype antibodies might also have a place in the treatment of B cell lymphoma/leukaemia (Hamblin *et al.*, 1980) was given encouragement in 1982 by a report by Miller *et al.* of a complete remission in a patient treated with a mouse anti-idiotype monoclonal antibody. It is disappointing that a similar response has not yet been observed in

**Figure 5** Karyotype and clone data of A31 tumour. Example of an ASG banded karyotype of clone A from a PWM stimulated spleen cell, 48 h culture.

*CLONE* $2n=39-y$ – 26 of the 42 metaphases karyotyped with $2n=39$ had the Y chromosome missing. The rest had a single chromosome loss which was not repetitive, and therefore did not constitute clones, and could be metaphases broken at the preparative stage.

$2n=40$ Df (2;2) – 6% of the $2n=40$ metaphases had a deletion of chromosome 2. (df: 2:2) The rest had an apparently normal karyotype.

$2n=41$ —— *Clone A* – the predominant clone at 89% of the $2n=41$ metaphases analysed.

*Clone A* Ts (2 with 2 Df 2:2) – Chromosome 2, trisomic with two chromosomes deleted at E.1. to F.3. M6 – Chromosome 6 – monosomy.

T (11; 4: MAR 1: 3) – Chromosome 11 – translocation of a segment, 4 to marker chromosome no. 1 at region 3. TS17 – Trisomy 17. –Y – Loss of chromosome Y.

MAR I/T (11: 4: MAR 1; 3) – Translocation of region 4 of chromosome 11 to region 3 of marker 1. (Rest of marker not identified.) Mar. 2 – Marker 2 not identified.

*Clone B* $2n=41$ TS 5 – *Clone B* $2n=41$, trisomic for chromosome 5, Y present 11% of cells analysed.

$2n=42$ – 2% of the population analysed. TS; 5, TS; 13 – Trisomic for chromosome 5 and 13. Y present.

Plus a small percentage of cells with abnormal chromosomes and aneuploidy (3%) which did not exist at a clonal level; i.e. minimum of three metaphases with the same karyotype.

No variations in clonal karyotype analysis was observed between the metaphases examined by the direct technique and by short term culture.

subsequent patients by these (Meeker *et al.*, 1985) or other workers (Rankin *et al.*, 1985). A number of research groups are examining closely the part that anti-idiotype antibodies may play in the therapy of lymphoma used alone (Lowder *et al.*, 1985), with attached drugs and toxins (Gilliland *et al.*, 1980; Embleton *et al.*, 1983), or carrying cell-sterilising amounts of radionuclides (Larsen *et al.*, 1983). In B cell lymphoma/leukaemia a major problem can arise in some patients from large quantities of circulating idiotype which can effectively block the entry of the therapeutic antibody into the tumour infiltrated tissues (Meeker *et al.*, 1985). It would seem therefore that successful treatment is more likely to be attained in patients with minimal levels of circulating idiotype.

The present tumour model, A31 in CBA/H mice, provides a close parallel to aggressive B cell lymphoma in man. As in man the A31 cells infiltrate aggressively the B cell domains of the lymphoreticular system and at a late stage the gonads and, less frequently, the central nervous system (CNS). The infrequency of terminal involvement of the CNS in the mouse might appear to be different from the situation in man where CNS involvement is a common problem. However, in man the CNS is usually only a clinical problem as a site of *relapse* following treatment. Overt CNS involvement in non-Hodgkin's lymphoma occurs in less than 10% of patients at diagnosis (Levitt *et al.*, 1980), and in acute lymphoid leukaemia CNS involvement is observed in less than 5% of children at diagnosis, and is rarely symptomatic (Bleyer, 1983).

We have included in this presentation the results of the response to drugs and X-radiation shown by mice with established B cell lymphoblastic lymphoma. There is clear response to a number of the drugs used in the therapy of this disease in man and also clear response to TBI, which is only rarely used in man for this disease.

The present work has shown that the A31 lymphoma is derived from the B cell lineage, expressing surface IgM with a $\kappa$ light chain and the Ia antigen. There was no evidence from immunofluorescence studies of the Thy 1.2. antigen found on T cells, or surface Ig of other isotypes. In particular we found no IgD which has been reported on other murine B cell lymphomas (Knapp *et al.*, 1979) and on the majority of human B cell leukaemias and lymphomas (Fu *et al.*, 1975). The A31 lymphoma was also negative for surface FcR and CR using the appropriately sensitised SRBC – although their presence on two other animal B cell tumours, $BCL_1$ (FcR) and $L_2C$ (CR), was confirmed in the present study (Shevach *et al.*, 1972; Knapp *et al.*, 1979). The absence of such receptors from the surface of A31 cells compared with $BCL_1$ and $L_2C$, is consistent with a tumour arrested at a different stage of B cell maturation (Rosenberg & Parish, 1977), or their loss during the process of tumour de-differentiation.

The secretion profile of IgM from A31 cells into the extracellular fluid was assessed during short-term culture *in vitro*. Immunoglobulin M was released over a 20 h period, but at levels which were consistent with a low secretor of Ig (Stevenson *et al.*, 1980*a*). Previous experience with B cell neoplasms has shown that this material represents small amounts of pentameric IgM released via a secretory pathway, rather than Ig turned over on the cell surface. Any contribution from the cell surface is most likely to occur as vesicle-bound Ig shed from the cells during culture (Stevenson *et al.*, 1980*a*). The absence of a detectable monoclonal band in the serum of tumour-bearing animals, even using sensitive techniques such as isoelectric focusing with immunoprecipitation, is also consistent with a low level of secretion.

A31 is potentially an eminently suitable model for immunotherapy investigations using anti-idiotype antibodies. Its abundant surface IgM with only minimal IgM secretion should ensure an almost unrestricted access of antibody to the tumour cells. Similar tumours described for the mouse, such as the $BCl_1$ of BALB/c, have often secreted significant levels of idiotypic IgM which provides a barrier to any therapeutic antibody (Tutt *et al.*, 1985). Production of anti-idiotype antibody for the A31 will probably require the isolation of the idiotypic IgM: in the present work idiotypic determinants associated with the whole tumour cell did not prove sufficiently immunogenic to generate a protective immune response. A number of techniques exist to provide this material (Stevenson & Glennie, 1985), but probably the most straight forward will be the production of a hybridoma between the A31 and a non-secreting mouse myeloma such as NS-1, which will secrete the tumour IgM. Armed with the 'rescued' IgM it becomes feasible to hyperimmunise animals for the production of both xenogeneic and syngeneic monoclonal anti-idiotype antibodies (Maloney *et al.*, 1985).

The cytogenetic studies showed that a characteristic of the dominant clone in the A31 tumour was deletion of an interstitial region of chromosome 2. Hayata *et al.* (1983) have reported deletion and loss of a segment of chromosome 2 in 44 of 52 cases of X-ray induced murine *myeloid* leukaemia. This change was seen irrespective of differences in mouse strain, sex and stage of tumour differentiation. This chromosome abnormality however was not observed in 30 cases of murine lymphoid leukaemia

(Hayata, unpublished data) nor in 9 cases of non-myeloid leukaemia examined in this laboratory. As far as we are aware this is the first observation of a chromosome 2 rearrangement in a non-myeloid leukaemia. The partial trisomy of chromosome 2 in A31 is a new observation as is the loss of chromosome 6, unless a part is translocated to either marker 1 or 2. Translocation of part of chromosome 11 is an uncommon event as is trisomy for 17. Variation in the presence or absence of the Y chromosome together with duplication is a common event in human and mouse myeloid leukaemia.

In summary, we are reporting the characteristics of a new murine B cell lymphoma which show that it is similar in many ways to aggressive B cell lymphoma in man. This would make it a useful model for the therapy of this disease and in addition it may be of value in expanding our knowledge of the biology of both normal and malignant B lymphocytes of mice and men.

We wish to thank Drs. A. Wild, A. Oliver and K. Moore for supplying various reagents. Miss S.A. Butler, Mrs D. Malowany, Miss C. Barker, Mr S. Humm and Mr J. Humphreys gave excellent technical assistance and Mr. D. Papworth carried out the statistical evaluation of the data. Dr. J.F. Loutit provided us with the tumour A31. The work in the Southampton laboratory was supported by Tenovus, The Cancer Research Campaign, the Medical Research Council and the Leukaemia Research Fund.

### References

BLEYER, W.A. (1983). Acute lymphoid leukaemia. *Pediatric Annals*, **12**, 277.

BRECKON, G. (1984). Brevans stain/mountant. *Mouse News Letter*, No. 69, p. 23.

COMMITTEE ON STANDARDISED GENETIC NOMENCLATURE FOR MICE. (1972). *J. Hered.*, **63**, 69.

EMBLETON, M.J., ROWLAND, G.F., SIMMONDS, R.G., JACOBS, E., MARSDEN, C.H. & BALDWIN, R.W. (1983). Selective cytotoxicity against human tumour cells by a vindesine-monoclonal antibody conjugate. *Br. J. Cancer*, **47**, 43.

ENGVALL, E. & PERLMANN, P. (1972). Enzyme-linked immunosorbent assay, ELISA. III. Quantitation of specific antibodies by enzyme-labeled anti-immunoglobulin in antigen-coated tubes. *J. Immunol.*, **109**, 129.

FORD, C.E. (1966). The use of chromosome markers. In: *Tissue Graft and Radiation*, Micklem & Loutit (eds.), p. 201. Academic Press: New York.

FU, S.M., WINCHESTER, R.J. & KUNKEL, H.G. (1975). Similar idiotypic specificity for the membrane IgD and IgM on human B lymphocytes. *J. Immunol.*, **114**, 250.

GALLIMORE, P.H. & RICHARDSON, C.R. (1973). An improved banding technique exemplified in the karyotype analysis of two strains of rat. *Chromosoma*, **41**, 259.

GILLILAND, D.G., STEPLEWSKI, Z., COLLIER, R.J., MITCHELL, K.F., CHANG, T.H. & KOPROWSKI, H. (1980). Antibody-directed cytotoxic agents: Use of monoclonal antibody to direct the action of toxin A chains to colorectal carcinoma cells. *Proc. Natl Acad. Sci.*, **77**, 4539.

GORDON, J. & ANDERSON, V.A. (1980). Isolation and characterisation of leukaemic B-lymphocytes: Influence of anticoagulant on C3-receptor detection, humoral killing and capping of cell surface immunoglobulin. *J. Immunol. Meth.*, **38**, 295.

GORDON, J. & STEVENSON, G.T. (1981). Antigenic modulation of lymphocyte surface immunoglobulin yielding resistance to complement-mediated lysis. II. Relationship to redistribution of the antigen. *Immunology*, **42**, 13.

HAMBLIN, T.J., ABDUL-AHAD, A.K., GORDON, J., STEVENSON, F.K. & STEVENSON, G.T. (1980). Preliminary experience in treating lymphocytic leukaemia with antibody to immunoglobulin idiotypes on the cell surfaces. *Br. J. Cancer*, **42**, 495.

HAYATA, I., SEKI, M., YOSHIDA, K., HIRASHIMA, K., SADO, T., YAMAGIWA, J. & ISHIHARA, T. (1983). Chromosomal aberration observed in 52 mouse myeloid leukaemias. *Cancer Res.*, **43**, 367.

JANOSSY, G. & GREAVES, M.F. (1971). Response of T and B lymphocytes to phytomitogens. *Clin. Exp. Immunol.*, **9**, 483.

KNAPP, M.R., JONES, P.P., BLACK, S.J., VITETTA, E.S., SLAVIN, S. & STROBER, S. (1979). Characterization of a spontaneous murine B cell leukemia ($BCL_1$) I. Cell surface expression of IgM, IgD, Ia and FcR. *J. Immunol.*, **123**, 992.

LARSON, S.M., CARRASQUILLO, J.A., KROHN, K.A. & 8 others. (1983). Localisation of $^{131}$I-labelled p97-specific Fab fragments in human melanoma as a basis for radiotherapy. *J. Clin. Invest.*, **72**, 2101.

LEVITT, L.J., DAWSON, D.M., ROSENTHAL, D.S. & MOLONEY, W.C. (1980). CNS involvement in the non-Hodgkin's lymphomas. *Cancer*, **45**, 545.

LOWDER, J.N., MEEKER, T.C. & LEVY, R. (1985). Monoclonal antibody therapy of lymphoid malignancy. *Cancer Surveys*, **4**, 359.

MALONEY, D.G., KAMINSKI, M.S., BUROWSKI, D., HAIMOVICH, J. & LEVY, R. (1985). Monoclonal anti-idiotype antibodies against the murine B cell lymphoma 38C13: characterization and use as probes for the biology of the tumour *in vivo* and *in vitro*. *Hybridoma*. **4**, 191.

MEEKER, T.C., LOWDER, J., MALONEY, D.G., MILLER, R.A., THIELEMANS, K., WARNKE, R. & LEVY, R. (1985). A clinical trial of anti-idiotype therapy for B cell malignancy. *Blood*, **65**, 1349.

MILLER, R.A., MALONEY, D.G., WARNKE, R. & LEVY, R. (1982). Treatment of B-cell lymphoma with monclonal anti-idiotype antibody. *N. Engl. J. Med.*, **306**, 517.

NADEL, E.M. (1977). History and further observations (1954–1976) on the $L_2C$ leukaemia in the guinea pig. *Fed. Proc.*, **36**, 2249.

RANKIN, E.M., HEKMAN, A., SOMERS, R. & HUININK, W.B. (1985). Treatment of two patients with B cell lymphoma with monoclonal anti-idiotype antibodies. *Blood*, **65**, 1373.

ROESS, D.A., RUH, T.S., BELLONE, C.J. & RUH, M.F. (1983). Glucocorticoid effects on lipopolysaccharide-stimulated murine B-cell leukemia line ($BCL_1$) cells. *Cancer Res.*, **43**, 2536.

ROSENBERG, Y.J. & PARISH, C.R. (1977). Ontogeny of the antibody-forming cell line in mice. IV. Appearance of cells bearing Fc-receptors, complement receptors, and surface immunoglobulin. *J. Immunology*, **118**, 612.

SHEVACK, E.M., ELLMAN, L., DAVIE, J.M. & GREEN, I. (1972). $L_2C$ guinea pig lymphatic leukemia: A 'B' cell leukemia. *Blood*, **39**, 1.

SKLAR, J., CLEARY, M.L., THIELEMANS, K., GRALOW, J., WARNKE, R. & LEVY, R. (1984). Biclonal B-cell lymphoma. *N. Engl. J. Med.*, **311**, 20.

STEVENSON, F.K., HAMBLIN, T.J., STEVENSON, G.T. & TUTT, A.L. (1980*a*). Extracellular idiotype immunoglobulin arising from human leukemic lymphocytes. *J. Exp. Med.*, **152**, 1484.

STEVENSON, F.K., MORRIS, D. & STEVENSON, G.T. (1980*b*). Immunoglobulin produced by guinea pig leukaemic B lymphocytes: its source and use as a monitor of tumour load. *Immunology*, **41**, 313.

STEVENSON, G.T. & GLENNIE, M.J. (1985). Surface immunoglobulin of B-lymphocytic tumours as a therapeutic target. *Cancer Surveys*, **4**, 213.

TUTT, A.L., STEVENSON, F.K., SLAVIN, S. & STEVENSON, G.T. (1985). Secretion of idiotypic IgM by the mouse B-cell leukaemia ($BCL_1$) occurs spontaneously *in vitro* and *in vivo*. *Immunology*, **55**, 59.